# Supplementary material for: The global burden of Klebsiella pneumoniae-associated lower respiratory infection in 204 countries and territories, 1990–2021: Findings from the global burden of disease study 2021
Source: PLoS One. 2025 May 19;20(5):e0324151. doi: 10.1371/journal.pone.0324151 (PMC12088015; doi:10.1371/journal.pone.0324151)
Supplement: Attachment — Submitted filename: rebuttal letter.docx [file pone.0324151.s004.docx]

Dear Reviewers and Editorial Team,

We sincerely appreciate the opportunity to revise our manuscript (PONE-D-24-54026) and thank you for your valuable feedback. We also greatly appreciate the time and effort the reviewers have dedicated to evaluating our manuscript. Their constructive comments have been invaluable in enhancing the clarity and quality of our study.

We have carefully addressed each of the editorial concerns and have made the necessary revisions as outlined below. For each comment, we provide a detailed response and describe the specific revisions made to the manuscript.

**Responses to the Editor's Comments:**

1. Compliance with PLOS ONE Formatting Requirements

Comment:

Please ensure that the manuscript meets PLOS ONE's style requirements.

Response:

We have reviewed and revised the manuscript to fully comply with PLOS ONE' s formatting guidelines. All headings, text formatting, and file naming conventions have been adjusted according to the provided templates. We have also moved the results content earlier in the manuscript, directly before the figures and tables, in accordance with PLOS ONE's guideline that each figure caption should appear immediately after the paragraph in which it is first cited, and tables should be included directly after their first citation.Additionally, we confirm that the author order is correct, with Juanjuan Li as the first author and Kaizhi Xu as the corresponding author. The corresponding author' s email address has been listed accordingly in the manuscript.

2. Funding Statement Correction

Comment:

Funding-related information should not be included in the Acknowledgments section.

Response:

We have removed funding-related text from the Acknowledgments section. Our Funding Statement remains unchanged:

"The author(s) received no specific funding for this work."

3. Data Availability Update

Comment:

The data repository previously used does not meet PLOS ONE' s standards.

Response:

To comply with PLOS ONE' s data availability requirements, we have uploaded the minimal dataset necessary to replicate our study' s findings to Figshare. The dataset is publicly accessible at the following DOI:https://doi.org/10.6084/m9.figshare.28616111,We have updated the Data Availability Statement in the manuscript accordingly.

4. ORCID iD Validation for Corresponding Author

Comment:

The corresponding author must have a validated ORCID iD in Editorial Manager.

Response:

The corresponding author's ORCID iD (0009-0005-2447-4491) has been successfully validated in Editorial Manager.

5. Copyright Compliance for Figures 3 and 4 (Map Images)

Comment:

Figures3 and 4 may contain copyrighted map images that must comply with the CC BY 4.0 license.

Response:

We confirm that the maps in Figures 3 and 4 were generated using the rnaturalearth R package, which provides access to Natural Earth map data. Natural Earth data is in the public domain and is not subject to copyright restrictions.

Data Source: Natural Earth is an open-source geographic dataset designed for unrestricted use, and further details can be found at https://github.com/ropensci/rnaturalearth.

Copyright Status: Natural Earth data is widely used for mapping and does not require explicit copyright permission.

Acknowledgment: If required, we are willing to include a statement in the figure legends acknowledging Natural Earth as the data source.

6. Reference List Verification and Formatting

Comment:

Ensure that the reference list is complete, correctly formatted, and free of retracted papers.

Response:

We have carefully reviewed the reference list to ensure accuracy and adherence to Vancouver style formatting. No retracted articles were found. Minor formatting inconsistencies have been corrected.

**Responses to the Reviewer' s Comments:**

Reviewer #1 Comments

Comment: The authors summarize the global epidemiological burden of lower respiratory infections (LRI) attributable to Klebsiella pneumoniae from 1990 to 2021, using data from the Global Burden of Disease Study. This reviewer considers this study analyze data that completely support the conclusions, with an appropriate statistical analysis. This analysis encompasses the incidence of Klebsiella infections in the respiratory tract in a broad and complete manner.

Response:

We sincerely thank the reviewer for the positive assessment of our study and for recognizing the robustness of our data analysis. We have carefully reviewed the manuscript to ensure clarity and coherence and have also implemented the necessary revisions based on Reviewer #2's comments.

Reviewer #2 Comments

1. Clarity and Readability of the Manuscript

Comment: The way the manuscript is presented is too confusing, and the wording is difficult to follow. Specific comments cannot be made because there is no numbering on each line of the manuscript, different font and paragraph styles are observed.

Response:

We appreciate the reviewer' s feedback regarding clarity and formatting. To improve readability, we have made the following modifications:

Ensured consistency in font and paragraph styles throughout the manuscript, adhering strictly to PLOS ONE formatting guidelines.

Revised complex and unclear sentences to improve readability.

Added line numbers to facilitate easier reference for future revisions.

These changes enhance the clarity and presentation of the manuscript.

2. Formatting Issues: Scientific Names and Abbreviations

Comment: Scientific names are misspelled (without italics), and there are inconsistencies in spacing. Tables contain abbreviations (e.g., ASR) that are not defined in the text.

Response:

We have carefully reviewed and corrected all instances of scientific names to ensure proper formatting:

Klebsiella pneumoniae is now consistently italicized throughout the manuscript.

All abbreviations (e.g., ASR, DALYs, SDI, EAPC) are now properly defined at their first mention in both the main text and table captions.

Inconsistent spacing issues have been addressed to align with the journal's formatting standards.

These corrections ensure accuracy and improve the manuscript' s professional presentation.

3. Refining Study Objectives

Comment:

The manuscript presents two objectives:

“This study aims to evaluate the trends and disparities at global, regional, and national levels, with a particular focus on Klebsiella pneumoniae.”

“This study aims to provide a comprehensive analysis of the epidemiology of Klebsiella pneumoniae-associated LRIs from 1990 to 2021.”

The second objective better defines the study, as the first one suggests an evaluation of trends in multiple bacterial species, while in reality, the study focuses only on Klebsiella pneumoniae.

Response:

We agree with the reviewer' s observation and have revised the study objective to ensure clarity:

The objective has been reworded to explicitly state that the study focuses on Klebsiella pneumoniae-associated LRIs rather than implying an evaluation of multiple pathogens.The relevant revision has been made in lines 51-55 of the introduction section.

4. Redundant Reporting of Results

Comment:

The manuscript presents overlapping results in different paragraphs, such as:

“…Conversely, the regions with the lowest age-standardized DALYs and death rates of LRI attributable to Klebsiella pneumoniae were Australasia, High-income North America, Eastern Europe, and East Asia…”

“… the most pronounced decreases in DALYs and death cases of LRI attributable to Klebsiella pneumoniae were observed in East Asia, whereas Southeast Asia, Southern Latin America, and Central Europe showed the most significant increases…”

Response:

We appreciate this observation and have carefully reviewed the Results section to eliminate redundancy. Specifically, we have:

Merged repetitive statements to present the findings more concisely.

Ensured that each paragraph presents unique information without unnecessary repetition.

These refinements improve the logical flow of the results, making the discussion more structured and easier to follow. The relevant modifications have been made in lines 231-233 of the Results section.

Final Remarks

We appreciate the reviewers' insightful feedback, which has significantly improved the clarity and quality of our manuscript. We have addressed all concerns and made the necessary revisions to enhance readability, formatting, and consistency.

We look forward to your further consideration of our revised manuscript. Please let us know if any additional modifications are required.

Best regards,
Kaizhi Xu
Zhejiang Provincial People's Hospital
